# Theophylline controllable RNAi-based genetic switches regulate expression of lncRNA TINCR and malignant phenotypes in bladder cancer cells

**DOI:** 10.1038/srep30798

**Published:** 2016-09-02

**Authors:** Zhicong Chen, Yuchen Liu, Anbang He, Jianfa Li, Mingwei Chen, Yonghao Zhan, Junhao Lin, Chengle Zhuang, Li Liu, Guoping Zhao, Weiren Huang, Zhiming Cai

**Affiliations:** 1 Key Laboratory of Medical Reprogramming Technology, Shenzhen Second People’s Hospital, The First Affiliated Hospital of Shenzhen University, Shenzhen 518039, Guangdong Province, People’s Republic of China; 2 Shantou University Medical College, Shantou 515041, Guangdong Province, People’s Republic of China; 3 Anhui Medical University, Hefei 230601, Anhui Province, People’s Republic of China; 4 Shanghai-MOST Key Laboratory of Health and Disease Genomics, Chinese National Human Genome Center at Shanghai, Shanghai 200000, Shanghai, China; 5 Department of Urology, Peking University First Hospital, Institute of Urology, Peking University, National Urological Cancer Centre, Beijing, 100034, China

## Abstract

TINCR is a well-known lncRNA which acts as a master regulator in somatic differentiation development. However, it is still unclear whether TINCR is also involved in caner occurrence and progression. In this study, we observed that TINCR was up-regulated in bladder cancer tissues and cells and contributed to oncogenesis and cancer progression. Silencing TINCR expression inhibited cell proliferation and promoted apoptosis *in vitro*, indicating that TINCR may be the potential therapeutic target for treating bladder urothelial carcinoma. Thus we used the synthetic biology approach to create theophylline controllable RNAi-based genetic switches which silenced TINCR in a dosage-dependent manner. Both RNAi-OFF and ON switches can be used to quantitatively control the expression of TINCR in bladder cancer to suppress the progression of bladder cancer. These findings suggest that lncRNA-TINCR could promote bladder cancer development and progression and artificial control of its expression through inducible RNAi may represent a new kind of therapeutic strategy for treating human bladder cancer.

The completion of the ENCODE project led to the staggering discovery that >80% of genome is transcribed into RNAs, whereas <3% is translated into proteins^1^,^2^. Such RNAs without protein- coding potential are referred to as noncoding RNAs (ncRNAs). LncRNAs, currently defined as endogenous non-coding transcripts greater than 200 nucleotides in length, have been found to perform a remarkable variety of biological functions^3^. Multiple studies have manifested that lncRNA have been implicated in different diseases, especially in cancers^4^. By influencing diverse cellular processes like proliferation, apoptosis, invasion and metastasis, cancer-associated lncRNAs have captured our great attention^5^,^6^,^7^. Terminal differentiation-induced ncRNA (TINCR), a lncRNA required for induction of key differentiation genes in epidermal tissue, is down-regulated in human squamous cell carcinoma^8^,^9^,^10^. In addition, TINCR is strongly up-regulated in human gastric carcinoma, where it was found to contribute to oncogenesis and cancer progression by regulating cell proliferation and apoptosis^11^. However, whether TINCR also acts as the hallmark of other cancers is still unknown and needed to be studied.

Bladder cancer (BCa) is the most common urologic malignancy with high prevalence and recurrence rates^12^,^13^. Although the prediction models of BCa have achieved a great progress in recent years, more novel biomarkers are still required for improving the specificity and reliability of prediction tools for BCa^14^. Besides, the mainstream therapies for BCa, such as surgery, radiation and chemotherapy, still perform limited accuracy and efficacy^15^,^16^,^17^. Thus, a better understanding of the molecular mechanisms involved in the pathogenesis of BCa and more attractive therapeutic approaches are definitely required. As mentioned above, the specific lncRNAs may provide novel therapeutic targets for the treatment of cancers. What’s more, growing studies have demonstrated that lncRNAs may serve as oncogenes or cancer suppressors in the BCa^6^. So we speculated that TINCR may also have function in BCa pathogenesis which is to be further researched.

Riboswitches are familiar as “functional noncoding RNA domains” that control associated gene expression by selectively binding metabolites and are involved in crucially regulatory networks at the transcriptional or post-transcriptional level in numerous types of bacteria^18^. There appears to be great interest in exploiting riboswitches for practical applications including gene therapy. Benefited from synthetic biology, these natural switches can be engineered to respond to any signal of choice and to regulate any gene of interest in the related expression platforms^19^. The versatility of designed riboswitches expands the tools available for genetics studies. Among them, theophylline aptamer, one of the most common and most widely utilized devices in the regulation of gene expression, can act as both OFF and ON switches to control protein expression in various platforms^20^,^21^,^22^,^23^. The theophylline-dependent miRNA or shRNA regulators which function as the “RNAi-OFF switches” were also highlighted in former studies^24^,^25^,^26^. However, there still lacks theophylline-controllable “RNAi-ON switches”.

Based on the engineering principles of medical synthetic biology, here, we presented the novel RNA regulators (theo-RNAi switches) which activate or inactivate the RNAi pathway to control TINCR expression in a theophylline dosage-dependent manner. In this study, we demonstrate that TINCR is up-regulated in BCa tissues and cell lines (SW780 and 5637) and contributes to oncogenesis and cancer progression. Silencing TINCR expression inhibits cell proliferation and promotes apoptosis *in vitro*. Theo-RNAi switches could be used to quantitatively control the expression of TINCR in BCa cell to suppress the progression of BCa.

## Results

### Expression of TINCR in BCa

**Figure 1:**
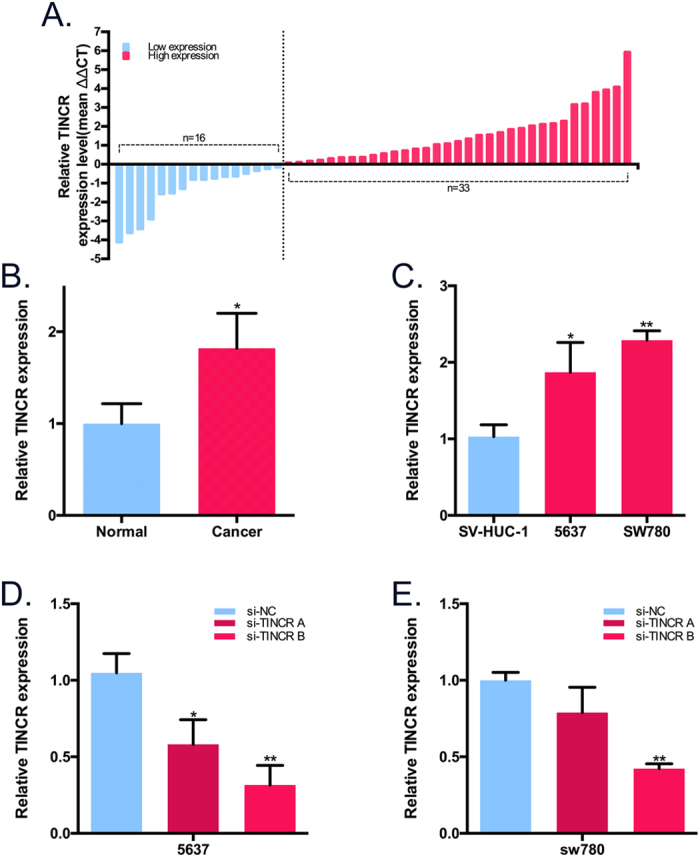
Expression of TINCR in BCa and expression change of TINCR after transfection of siRNAs performed by real-time qPCR. (**A**) Expression of TINCR expression was examined in 49 paired human BCa tissues and corresponding noncancerous tissues. Data are represented as ΔΔCt which are the normalized real-time qPCR cycle threshold (Ct) values of TINCR between cancer tissues and pair-matched noncancerous tissues, and defined as “<0” for low expression (n = 16) and “>0” for high expression (n = 33). (**B**) The relative expression level of TINCR was significantly higher in bladder cancer tissues compared with matched normal tissues (paired T test). Bar: mean ± SEM; *P < 0.05. (**C**) Compared with SV-HUC-1, the relative expression level of TINCR was overexpress significantly in BCa cell lines 5637 and SW780. Bars: mean ± SD; *P < 0.05, **P < 0.01. (**D,E**) TINCR expression level following the treatment of 5637 and SW780 cells with siRNAs. The si-TINCR B could significantly knock down TINCR in both 5637 and SW780. Bars: mean ± SD; *P < 0.05, **P < 0.01.

The relative expression level of TINCR was measured by real-time qPCR in 49 pair-matched specimens obtained from patients with BCa and 2 BCa cell lines. Overexpression of TINCR was observed in BCa tissues compared with paired non-cancerous tissues (Fig. 1A,B). Additionally, advanced TNM stage had a positive relationship with up-regulated expression of TINCR (Table 1). But the other parameters showed no correlation with TINCR expression level. Furthermore, real-time PCR analysis demonstrated that TINCR was expressed at a higher level in 2 BCa cell lines, 5637 (P < 0.05) and SW780 (P < 0.01), compared to the normal bladder cell line SV-HUC-1 (a SV-40-immortalized human uroepithelial cell line) (Fig. 1C). 49 patients’ clinical characteristics are shown in Table 2. Together, these data suggested that TINCR may play an oncogenic role in BCa.

### Effects of TINCR on the proliferation and apoptosis of BCa cells *in vitro*

To test the hypothesis that TINCR functions as an oncogene in BCa, we down-regulated endogenous TINCR expression in 2 BCa cell lines 5637 and SW780 using chemically synthesized siRNAs. To ensure the RNAi efficiency in target regulation, we synthesized two different siRNAs (si-TINCRs A and B) according to the former study^9^ and si-TINCR B was considered appropriate for knockdown of TINCR. Compared with the negative control siRNA, the relative expression level of TINCR in 5637 and sw780 was significantly down-regulated by si-TINCR B (Fig. 1D,E).

**Figure 2:**
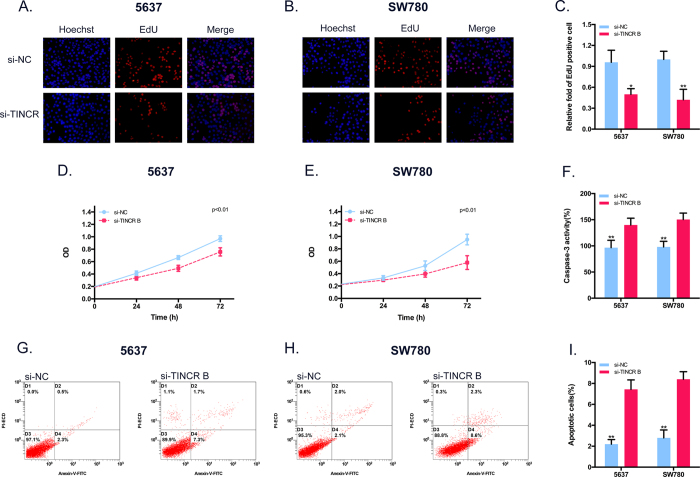
Effects of TINCR on the proliferation and apoptosis of BCa cells *in vitro*. (**A,B**) Representative images of EdU assay in BCa cell after transfection siRNAs. (**C**) EdU incorporation rate was manifested as ratio of EdU positive cells relative to Hoechst 33342 positive cells. Silencing TINCR could activate the proliferation inhibition of BCa cell. Bars: mean ± SD; **P < 0.01. (**D,E**) BCa cell growth inhibition was observed after transfection with si-TINCR detected by CCK-8 assay at 0, 24, 48 and 72 h. P < 0.01. (**F**) Decreasing TINCR promoted the activity of caspase-3 in BCa cells. Bars: mean ± SD; **P < 0.01. (**G,H**) Representative scatter plots of flow cytometry assay in BCa cell after transfection siRNAs. (**I**) Down-regulating TINCR increased apoptosis ratio in BCa cells. Bars: mean ± SD; **P < 0.01.

We further determined whether TINCR promotes cell proliferation in BCa. After cells were transiently transfected with si-TINCR and si-NC, EdU assay (Fig. 2A–C) and CCK-8 assay (Fig. 2D,E) were performed and the results revealed that knockdown of TINCR suppressed cell growth in 2 Bca cell lines. Meanwhile, to further investigate the effect of TINCR on the apoptosis of BCa cells, we performed flow cytometric assay and caspase 3 ELISA assay. The results demonstrated that the activities of caspase 3 and the apoptotic cells were significantly increased among the siTINCR-transfected cells as compared with the si-NC-treated cells. (Fig. 2F–I). All these data suggested that silencing TINCR inhibits cell growth and induces apoptosis in BCa.

### Construction of theo-RNAi switches

To further expand on this issue and to more precisely control the expression of TINCR, we set out to construct the theo-RNAi switches targeting TINCR based on the engineering principles of synthetic biology. The theo-RNAi switches can be turned off or turned on through binding of theophylline.

**Figure 3:**
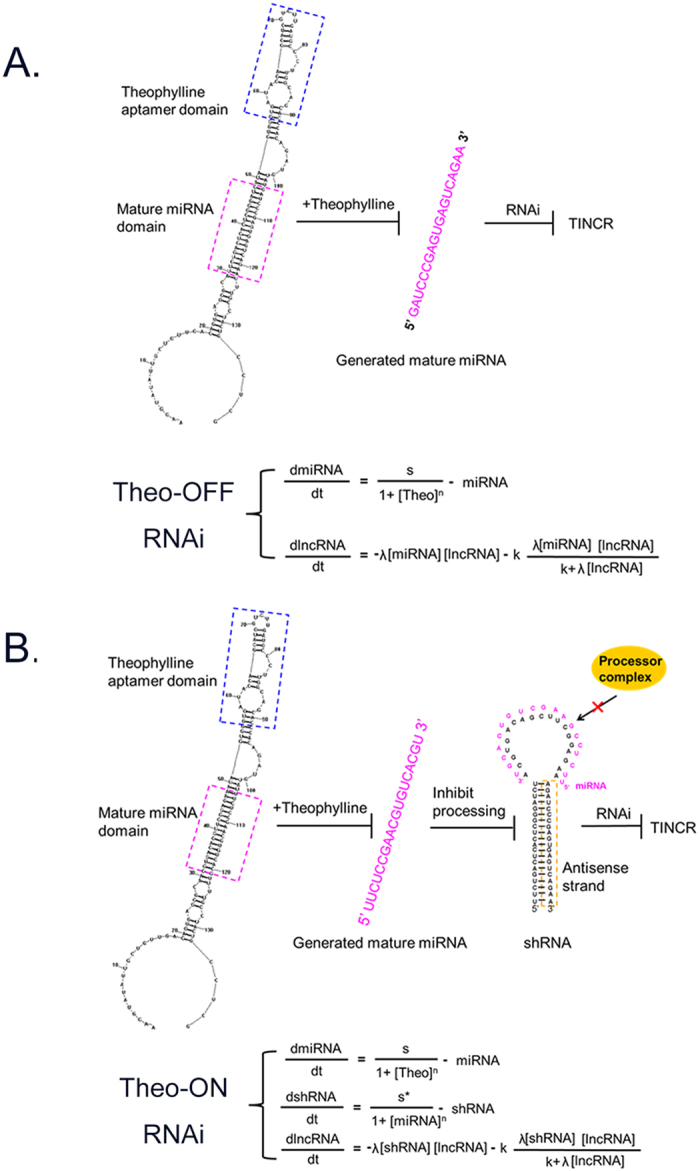
Design and characterization of theophylline controllable RNAi-based genetic switches. (**A**) OFF device: RNAi inhibition by theophylline was obtained when the RNA aptamer for theophylline was incorporated into the loop region of the artificial miRNA. Secondary structures of the RNA transcript, aptamer domain, mature miRNA domain and equations describing separate processes were shown. (**B**) ON device: The artificial miRNA controlled by theophylline was designed to inhibit the processing of shRNA silencing TINCR. Secondary structures of the pre-miRNA and pre-shRNA, aptamer domain, mature miRNA domain and equations describing separate processes were also shown. s, the normal expression rate of miRNA or shRNA in physiological status. n, inhibitory parameter of RNAi.-miRNA or -shRNA, the degradation rate of miRNA or shRNA in cell. λ, affinity of the miRNA or shRNA target site for the RISC complex. K, lncRNA cleavage rate of bound RISC. [x], the concentration of x.

The first and foremost, we constructed the theo-dependent RNAi-OFF switch that transcribed a pre- artificial miRNA in which the RNA aptamer for theophylline was inserted into the loop region of the pre- miRNA scaffold. The stem region of this scaffold was the sequence that encoded mature artificial miRNA targeting TINCR. The design for fusing RNA aptamer was inspired by the observations that some ligand regulators can selectively recognize the terminal loops of miRNA precursors, prevent the cleavage processes from Microprocessor complex and inhibit the generation of mature miRNA sequence^27^,^28^. So in the RNAi-OFF switch, the RNAi inhibition and TINCR derepression would be induced by the addition of theophylline (Fig. 3A).

Then we constructed the theo-dependent RNAi-ON switch by connecting the RNAi-OFF switch and a shRNA targeting TINCR. The mature miRNA was designed to pair with loop region of the shRNA scaffold. This design was also inspired by the stereo-hindrance effect, where the existence of a miRNA binding site in a pre-shRNA region suppressed the processing of the transcript. So in the RNAi-ON switch, the RNAi activation and TINCR repression would be induced by the addition of theophylline (Fig. 3B).

At last, we developed mathematical models to inform the design of two devices to elucidate important parameters. The models indicated the relationships between the theophylline concentration and lncRNA knockdown efficiency using a series of differential equations describing the each process. The model predicted that increasing the input concentration would lead to substantial increases in the change in target gene expression, and that the relationship was nonlinear and saturable, which were the key features of many biological phenomena.

### Effects of theo-RNAi switches on the expression of TINCR *in vitro*

**Figure 4:**
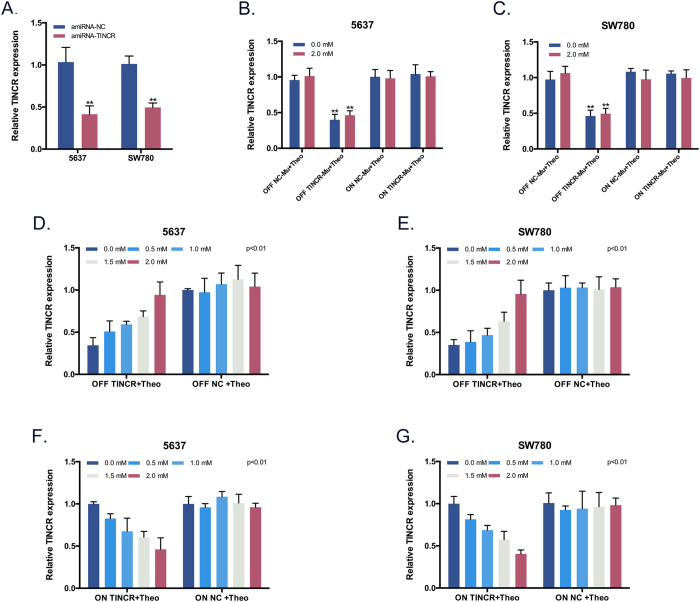
Validation of theo-RNAi switches of BCa cells. (**A**) TINCR expression level following the treatment of BCa cells with amiRNAs. The amiRNA-TINCR could significantly knock-down TINCR in both 5637 and SW780. Bars: mean ± SD; **P < 0.01. (**D,E**) TINCR expression level following the treatment of BCa cells with the OFF device at varying concentration of theophylline. Bars: mean ± SD; P < 0.01. (**F,G**) And the ON device. Bars: mean ± SD; P < 0.01. (**B,C**) TINCR expression level following the treatment of BCa cells with the mutant theo-RNAi switches at 0/2 mM theophylline. Bars: mean ± SD; **P < 0.01.

To test whether the behaviors of the switches were consistent with theory predictions, we set up various ranges of theophylline concentrations according to relative researches^20^,^21^,^22^,^23^. First and foremost, the effect of varying concentrations of theophylline (0–4 mM) on cell viability was measured by CCK8 assay and the influence of theophylline on the TINCR expression was detected by real-time qPCR. We found that 0–2 mM theophylline have minimal effects on TINCR expression and cell viability. While the expression of TINCR was still unaffected by 4 mM theophylline, a moderate growth inhibition of BCa cells was observed at this concentration (Supplement-1). Then we explored the function of the artificial miRNA targeting TINCR by real-time qPCR. Compared with the negative control miRNA, the relative expression level of TINCR in BCa cell was significantly down-regulated by amiRNA-TINCR (Fig. 4A). After transfection of the RNAi-OFF switch, RNAi-ON switch and corresponding negative control device, respectively, the results showed that these theophylline-regulated RNAi systems quantitatively regulated the expression of TINCR in both two switches at 0–2 mM theophylline, and that 2 mM theophylline caused the greatest induction (Fig. 4D–G). We therefore carried out further experiments at 2 mM theophylline. To further confirm that the engineered theo-RNAi systems performed functions through using theophylline aptamer, we constructed other theo-RNAi systems (negative controls) which contained mutant aptamers. The data indicated that theo-RNAi-NC systems lost the abilities to respond to theophylline (Fig. 4B,C).

### Effects of theo-RNAi switches on the proliferation and apoptosis of BCa cells *in vitro*

To evaluate the utility of theo-RNAi switches in BCa cells, we repeated the functional assays. BCa cells 5637 and SW780 cells were transfected with theo-RNAi switches and cultured at 0 or 2 mM theophylline, respectively.

**Figure 5:**
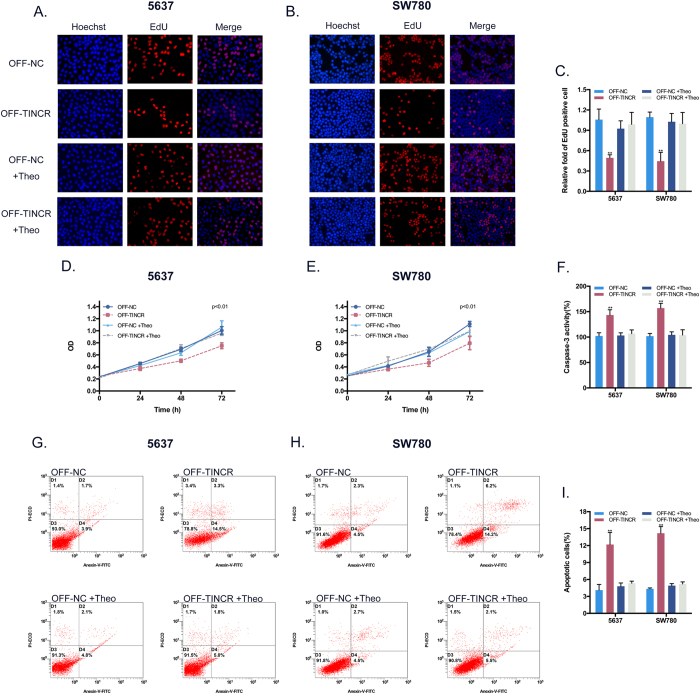
Effects of the OFF device on the proliferation and apoptosis of BCa cells *in vitro*. (**A,B**) Representative images of EdU assay in BCa cell after transfection the OFF device. (**C**) EdU assay manifested that the proliferation inhibition of BCa cell activated by silencing TINCR could be switched off by the OFF device at 2 mM theophylline. Bars: mean ± SD; **P < 0.01. (**D,E**) CCK8 assay demonstrated that growth inhibition of BCa cell activated by silencing TINCR could be turned off by the OFF device at 2 mM theophylline. P < 0.01. (**F**) ELISA assay supported that the activity of caspase-3 activated by silencing TINCR could be turned off by the OFF device at 2 mM theophylline. (**G,H**) Representative scatter plots of flow cytometry assay in BCa cell after transfection the OFF device. (**I**) Flow cytometry assay showed that the OFF device at 2 mM theophylline could turn off the promotion of apoptosis activated by silencing TINCR. Bars: mean ± SD; **P < 0.01.

Using RNAi-OFF switch, the results of EdU assay (Fig. 5A–C) and CCK8 assay (Fig. 5D,E) indicated that cell growth was significantly inhibited at 0 mM theophylline compared with the concentration at 2 mM. Meanwhile, the number of apoptotic cells was also significantly increased at 0 mM as revealed by ELISA assay (Fig. 5F) and flow cytometric assay (Fig. 5G–I). These findings confirmed that TINCR inhibits cell growth and induces apoptosis in BCa and that RNAi can be “turned off” due to the RNAi-OFF switch.

**Figure 6:**
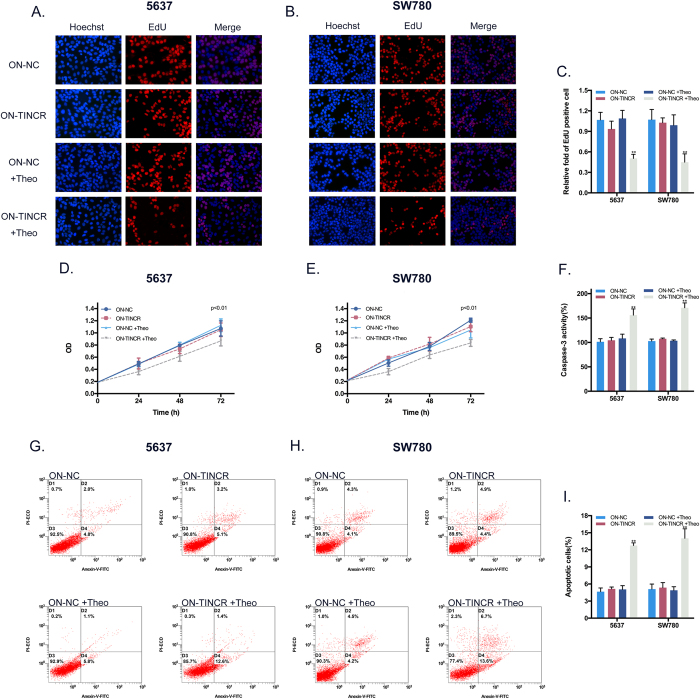
Effects of the ON device on the proliferation and apoptosis of BCa cells *in vitro*. (**A,B**) Representative images of EdU assay in BCa cell after transfection the ON device. (**C**) EdU assay manifested that the proliferation inhibition of BCa cell activated by silencing TINCR could be turned on by the ON device at 2 mM theophylline. Bars: mean ± SD; **P < 0.01. (**D,E**) CCK8 assay demonstrated that growth inhibition of BCa cell activated by silencing TINCR could be switched on at 2 mM theophylline by the ON device at 2 mM theophylline. P < 0.01. (**F**) ELISA assay supported that the activity of caspase-3 activated by silencing TINCR could be turned on by the ON device at 2 mM theophylline. (**G,H**) Representative scatter plots of flow cytometry assay in BCa cell after transfection the ON device. (**I**) Flow cytometry assay showed that the ON device at 2 mM theophylline could turn on the promotion of apoptosis activated by silencing TINCR. Bars: mean ± SD; **P < 0.01.

Oppositely, the expression of TINCR can be “switched off” at 2 mM theophylline by RNAi-ON switch. EdU assay (Fig. 6A–C) and CCK8 assay (Fig. 6D,E) demonstrated that BCa cells proliferation was decreased by 2 mM theophylline. In the meantime, flow cytometric assay (Fig. 6G–I) and ELISA assay (Fig. 6F) indicated that the apoptotic level of BCa cells were increased by 2 mM theophylline. Together, theo-RNAi switches targeting TINCR provided an effective platform for precisely regulation of proliferation and apoptosis in BCa cells through a dosage-dependent manner.

## Discussion

An emerging revolution has been sparked in the lncRNA field due to the novel technologies. In almost all aspects of biology, lncRNAs have served as critical regulators^29^,^30^. Accumulating evidences suggest that lncRNAs play functional roles in development of different cancers, including cancer initiation and progression^30^. Also, increasing findings from basic studies to clinical researches have emphasized that lncRNAs are involved in BCa^6^. For example, H19, the oldest distinguished lncRNA, is overexpressed and induces cell proliferation, metastasis, and survival in BCa^31^,^32^,^33^. Recently, the further comprehensive mechanism between H19 and BCa was reported in continuance^34^,^35^. Our previous researches also identified several lncRNAs, like PVT1 and SUMO1P3, which play oncogenic roles in BCa^36^,^37^. Therefore, the identification of BCa-associated lncRNAs may provide an opportunity to reveal the mechanism network of cancer pathogenesis in BCa for further targeted treatment.

In this study, we found that TINCR expression was significantly up-regulated in BCa tissues and cells. Also, patients with higher TINCR levels presented the higher tumor stage. Thus, we highly speculated TINCR may also function in BCa. To further test this hypothesis, a series of functional tests were performed in 2 BCa cell lines 5637 and SW780. More assays than one were taken in every aspect in order to reduce errors. CCK8 assays and EdU assays revealed that silencing TINCR expression contributed to significant inhibition of BCa cell proliferation. Flow cytometric assay and ELISA assay demonstrated that knockdown of TINCR expression led to promotion of BCa cell apoptosis. Taken together, our data revealed that TINCR might serve as a potential oncogene in BCa. Unfortunately, the further explorations of the underlying biological mechanisms haven’t been done due to the restriction of experimental conditions.

With the remarkable development of synthetic biology, the therapeutic strategies of diseases have been improved with each passing day^38^. Versatile synthetic gene circuits have been designed and constructed for the treatment of various diseases, such as metabolic disorders and cancers^39^,^40^,^41^. Inspired by these notable works, we created tetracycline-inducible shRNA targeting one oncogenic lncRNA to inhibit cell growth and induce apoptosis in bladder cancer cells in one of our published studies^34^. However, tetracycline is not an ideal inducer due to its mitochondrial toxicity. We still need to develop RNAi-based genetic switches based on non-toxic inducers. Theophylline is such a ligand which is relatively nontoxic and cell-permeable. In previous works^22^,^23^,^24^, there are two general ways for constructing theophylline controllable RNAi regulators. One type of these regulators is constructed by coupling a theophylline RNA aptamer to the loop region of a shRNA or to the basal segment of a pre-miRNA. Binding of the ligand to the aptamer domain sterically inhibits RNAi biogenesis by Dicer or Drosha. Another type of these regulators utilizes a strand displacement strategy depend on ligand binding events to reduce siRNA production. In contrast to the two traditional switches which could only achieve RNAi-OFF function^22^,^23^,^24^, we constructed the novel system which could function as both RNAi-OFF and RNAi-ON switches in this work. Benefited from the theo-RNAi switches targeting TINCR, the expression of TINCR could be quantitively regulated in a certain concentration range. The experimental results were well consistent with the predictions of mathematical equations. The malignant phenotypes of BCa influenced by TINCR could also be turned off or turned on by the RNAi-OFF and ON switches. These novel molecular devices would greatly advance our cellular engineering abilities for a variety of biological applications in medicine, such as cell therapy, immunotherapy and cancer gene therapy. One notable problem is that cellular expression of the theo-RNAi system was still unstable because of the low transient transfection efficiency. Packing this system with lentiviral vectors and constructing stable cell lines may highly improve the transfection efficiency and heterologous expression. Another notable problem is that the reported shRNA-based RNAi regulator^25^ only worked when the aptamer was placed in very close proximity to the siRNA. However, our theo-RNAi system which contains several remained bases between the mature miRNA and aptamer still effectively responded to varying concentrations of theophylline. Since the sequence of theophylline aptamer used in this work is somewhere different from the aptamer for constructing shRNA-based RNAi regulator, a possible explanation is that the theophylline-binding site was also changed due to the varied secondary structures of the RNA aptamers. Future works should test and compare the efficiencies of the various theo-RNAi systems in which aptamers were inserted at different places.

In conclusion, our findings demonstrated that knockdown of lncRNA-TINCR could inhibit the malignant phenotypes of bladder cancer cells, and TINCR may be used as a potential therapeutic target for bladder cancer. Besides, the theo-RNAi-TINCR switches present a specific useful tool to inhibit un-controlled growth of BCa cells by controlling the expression of TINCR.

## Materials and Methods

### Patient samples and clinical data collection

Tumor samples were collected during the surgery, and non-tumor samples were cut at least 5 cm away from the tumors. A total of 49 paired BCa tissues and corresponding noncancerous tissues were included in this study. The methods were carried out in accordance with the approved guidelines. Written informed consent was obtained from all subjects. All experimental protocols were approved by the Research Ethics Committee of Shenzhen Second People’s Hospital.

### Cell lines and cell culture

BCa cells (5637, SW780) and SV-40-immortalized human uroepithelial cell line (SV-HUC-1) were obtained from the Institute of Cell Research, Chinese Academic of Sciences, Shanghai, China. 5637 cells were maintained in RPMI-1640 media; SW780 cells were maintained in DMEM media; SV-HUC-1 cells were maintained in F12K medium in a humidied atmosphere of 5% CO_2_ maintained at 37 °C. All the media were also supplemented with 10% fetal bovine serum (FBS) and 1% penicillin-streptomycin.

### Plasmids, siRNAs and transfection

All siRNAs and plasmids involved in this study were synthesized by GenePharma, Shanghai, China. The sequences of siRNA oligonucleotides were listed as follow: si-TINCR A (sense sequence): 5′-GCAUGAAGUAGCAGGUAUUUU-3′; si-TINCR B (sense sequence):5′-GAUCCCGAGUGAGUCAGAAUU-3′; si-NC (sense sequence): 5′-UUCUCCGACGUGUCACGUTT-3′. The sequences of the theo-RNAi switches and the negative control system were designed and chemically synthesized. All these related sequences were inserted into siCHECK™-2 vector at the restriction sites of Xhol/Notl and pGPU6-GFP-Neo at the restriction sites of BbsI/BamHI, respectively. Cells were seeded in the plates till they reached 50–70% confluency and transiently transfected with the mixtures of siRNAs or plasmids using Lipofectamine® 3000 (Life Technologies) according to the manufacturer’s instructions.

### RNA extraction and real-time qPCR analysis

Total RNA from tissues or cells was isolated with Trizol reagent (Life Technologies) and quantified by NanoDrop according to the manufacturer’s instructions. cDNA was synthesized using the PrimeScript RT Reagent Kit with gDNA Eraser (Takara) according to the manufacturer’s instructions. Real-time qPCR was performed using the SYBR Premix Ex Taq II (Takara) with the follow primers. TINCR primers forward: 5′-TGTGGCCCAAACTCAGGGATACAT-3′, reverse: 5′-AGATGAC AGTGGCTGGAGTTGTCA-3′; GAPDH primers forward: 5′-CGCTCTCTGCTCCTCCTGTTC-3′, reverse: 5′-ATCCGTTGACTCCGACCTTCAC-3′. The 2^−ΔCt^ method was used to calculate the relative amount of TINCR compared with GAPDH expression.

### Cell proliferation assays

Cell proliferation assays were performed by the Cell Counting Kit-8, CCK-8 (Beyotime Institute of Biotechnologya) and ethynyl-2-deoxyuridine, EdU kit (Ribobio) according to the manufacturer’s instructions. Both of the two experiments were performed as previously reported in Zhan *et al.*^37^. The assays were performed in triplicate and the each experiments were repeated three times.

### Cell apoptosis assays

Cell apoptosis assays were detected by the flow cytometry and the caspase-3 ELISA kit (Cusabio) according to the manufacturer’s protocols. Both of the two experiments were also performed as previously reported in Zhan *et al.*^37^. Each experiment was repeated at three times.

### Statistical analysis

All statistical analyses were executed by using SPSS 22.0 software (IBM). Data were presented as mean ± SD, except for Fig. 1B which used mean ± SEM. Statistical significance was determined by Student’s 2-tailed t-test or ANOVA. In all cases, P < 0.05 was considered statistically significant. *p < 0.05; **p < 0.01.

## Additional Information

**How to cite this article**: Chen, Z. *et al.* Theophylline controllable RNAi-based genetic switches regulate expression of lncRNA TINCR and malignant phenotypes in bladder cancer cells. *Sci. Rep.* **6**, 30798; doi: 10.1038/srep30798 (2016).

## Supplementary Material

Supplementary Information

## Figures and Tables

**Table 1:** Correlation between TINCR expression and clinicopathological characteristics of bladder cancer patients.

Parameters	Group	Total	TINCR expression		P value
			High	Low	
Gender	Male	37	26	11	0.492
	Female	12	7	5	
Age (years)	<59	14	10	4	1.000
	≥59	35	23	12	
Tumor size (cm)	<3	20	11	9	0.215
	≥3	19	22	7	
Histological grade	L	18	10	8	0.211
	H	32	24	8	
TNM stage	0/I	18	8	10	0.035*
	II/III/IV	31	25	8	
Lymph nodes metastasis	N0	47	31	16	1.000
	N1 or above	2	2	0	

^*^P < 0.05 was considered significant (Chi-square test between 2 groups).

**Table 2:** Summary of clinicopathological features of tissues of bladder cancer.

Pt No.	Sex	Age	Grade	Stage	Pt No.	Sex	Age	Grade	Stage
1	M	76	H	T2N0M0	26	M	68	L	T2bN0M0
2	M	66	L	T1N0M0	27	M	59	L	T2bN0M0
3	F	41	L	T1N0M0	28	M	51	H	T2N0M0
4	M	59	H	T4N0M0	29	M	63	H	T2bN0M0
5	F	80	H	T2N0M0	30	M	70	L	T2aN0M0
6	F	74	H	T3aN0MO	31	F	73	H	T2aN0M0
7	M	75	H	T2bN0M0	32	M	74	H	T3N0M0
8	M	69	L	T1NxM0	33	M	57	H	T4aN0MO
9	M	63	H	T1NxM0	34	M	45	L	TaN0M0
10	M	77	H	T1N0M0	35	F	61	H	T3aN0MO
11	M	59	L	T1N0M0	36	M	52	L	T1N0M0
12	M	64	H	T3aN0MO	37	M	65	H	T2bN0M0
13	M	53	L	T1N0M0	38	M	58	H	T4aNOMO
14	M	48	L	TaN0M0	39	M	62	H	T4aN0M0
15	F	56	L	T1N0M0	40	M	66	H	T2bN2M0
16	F	62	L	T1N0M0	41	F	89	H	T2bN0M0
17	M	60	L	T2N0M0	42	F	72	H	T3N0M0
18	M	57	H	T2N0M0	43	M	65	L	T1N0M0
19	M	71	H	T2N0M0	44	M	74	H	T2bN0M0
20	F	75	H	T1N0M0	45	M	68	H	T3N0M0
21	M	58	H	T4aN3MO	46	F	64	H	T1N0M0
22	F	47	H	T2N0M0	47	M	54	H	T1N0M0
23	M	84	L	T3N0M0	48	M	68	L	TaN0M0
24	M	53	L	T1N0M0	49	M	78	H	T2aN0M0
25	M	63	H	T3aN0MO					

Pt No. -patient number; M male; F female; Grade-the World Health Organization 2004 classification: H high; L low; Stage-the American Joint Committee on Cancer TNM classification.
