# Author Correction: Theophylline controllable RNAi-based genetic switches regulate expression of lncRNA TINCR and malignant phenotypes in bladder cancer cells

**DOI:** 10.1038/s41598-023-49416-3

**Published:** 2023-12-21

**Authors:** Zhicong Chen, Yuchen Liu, Anbang He, Jianfa Li, Mingwei Chen, Yonghao Zhan, Junhao Lin, Chengle Zhuang, Li Liu, Guoping Zhao, Weiren Huang, Zhiming Cai

**Affiliations:** 1 Key Laboratory of Medical Reprogramming Technology, Shenzhen Second People’s Hospital, The First Affiliated Hospital of Shenzhen University, Shenzhen, 518039 Guangdong Province People’s Republic of China; 2 Shantou University Medical College, Shantou, 515041 Guangdong Province People’s Republic of China; 3 Anhui Medical University, Hefei, 230601 Anhui Province People’s Republic of China; 4 Shanghai-MOST Key Laboratory of Health and Disease Genomics, Chinese National Human Genome Center at Shanghai, Shanghai, 200000 Shanghai China; 5 Department of Urology, Peking University First Hospital, Institute of Urology, National Urological Cancer Centre, Peking University, Beijing, 100034 China

Correction to: *Scientific Reports* 10.1038/srep30798, published online 02 September 2016 

This Article contains errors in Figure 6, where the grouping of data points within the ‘5637 ON-NC’ subgroup in panels G and I is incorrect. The correct Figure 6 and accompanying legend appear below.

**Figure 6:**
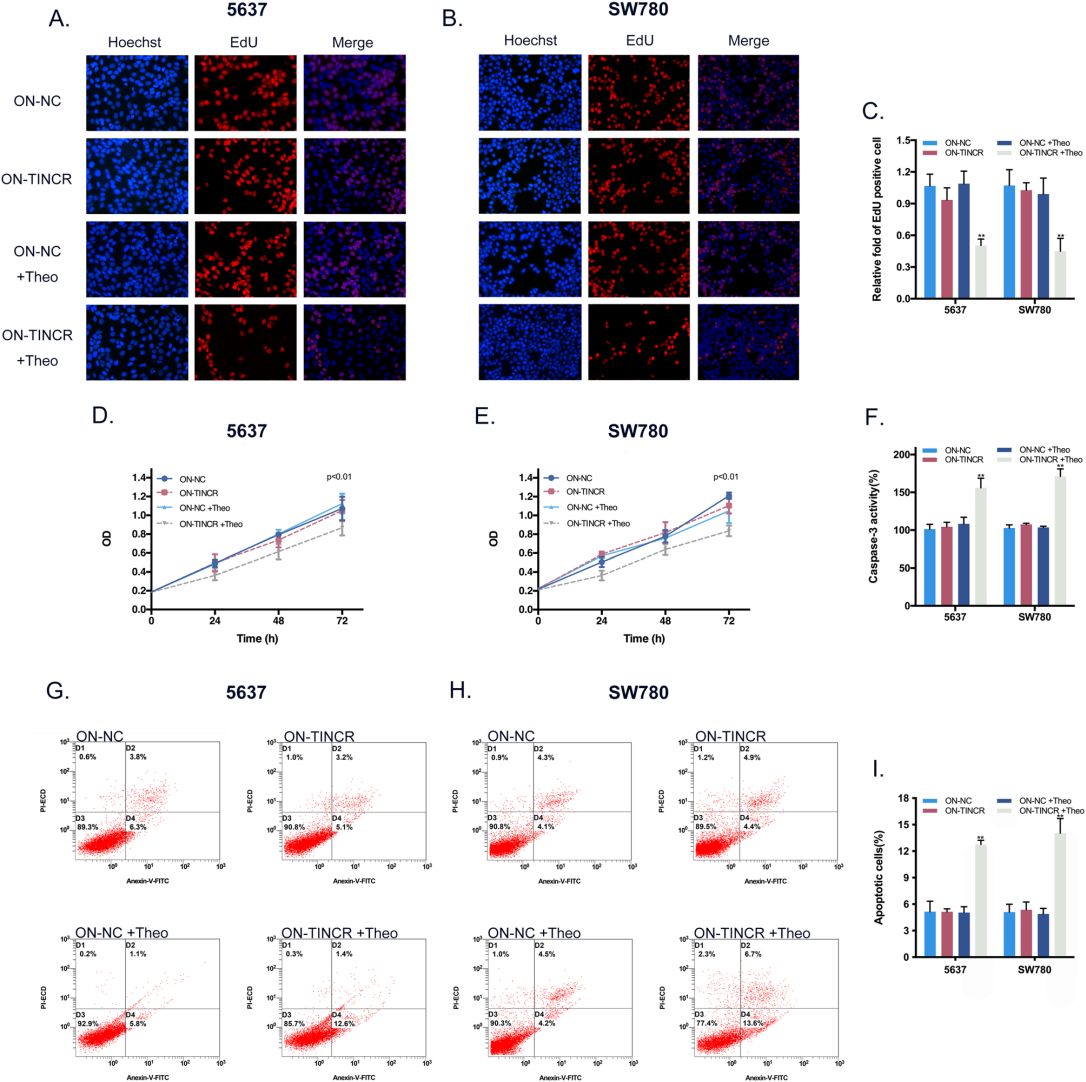
Effects of the ON device on the proliferation and apoptosis of BCa cells in vitro. (**A, B**) Representative images of EdU assay in BCa cell after transfection the ON device. (**C**) EdU assay manifested that the proliferation inhibition of BCa cell activated by silencing TINCR could be turned on by the ON device at 2 mM theophylline. Bars: mean ± SD; **P < 0.01. (**D, E**) CCK8 assay demonstrated that growth inhibition of BCa cell activated by silencing TINCR could be switched on at 2 mM theophylline by the ON device at 2 mM theophylline. *P* < 0.01. (**F**) ELISA assay supported that the activity of caspase-3 activated by silencing TINCR could be turned on by the ON device at 2 mM theophylline. (**G, H**) Representative scatter plots of flow cytometry assay in BCa cell after transfection the ON device. (**I**) Flow cytometry assay showed that the ON device at 2 mM theophylline could turn on the promotion of apoptosis activated by silencing TINCR. Bars: mean ± SD; **P < 0.01.

