# Supplementary material for: Theophylline controllable RNAi-based genetic switches regulate expression of lncRNA TINCR and malignant phenotypes in bladder cancer cells
Source: Sci Rep. 2016 Sep 2;6:30798. doi: 10.1038/srep30798 (PMC5009373; doi:10.1038/srep30798)
Supplement: Supplementary Information [file srep30798-s1.doc]

Theophylline controllable RNAi-based genetic switches regulate expression of lncRNA TINCR and malignant phenotypes in bladder cancer cells

Zhicong Chen^1, 2†^, Yuchen Liu^1†^, Anbang He^1, 3†^, Jianfa Li^1^, Mingwei Chen^1^, Yonghao Zhan^1^, Junhao Lin^1^, Chengle Zhuang^1^, Li Liu^1^, Guoping Zhao^4^, Weiren Huang^1^, Zhiming Cai^1, 5^

^1^Key Laboratory of Medical Reprogramming Technology, Shenzhen Second People’s Hospital, The First Affiliated Hospital of Shenzhen University, Shenzhen 518039, Guangdong Province, People’s Republic of China

^2^Shantou University Medical College, Shantou 515041, Guangdong Province, People’s Republic of China

^3^Anhui Medical University, Hefei 230601, Anhui Province, People’s Republic of China

^4^Shanghai-MOST Key Laboratory of Health and Disease Genomics, Chinese National Human Genome Center at Shanghai, Shanghai 200000, Shanghai, China

^5^Department of Urology, Peking University First Hospital, Institute of Urology, Peking University, National Urological Cancer Centre, Beijing, 100034, China.

†Equal contributors

Correspondence to: Zhiming Cai, email: caizhiming2000@163.com

Weiren Huang, email: pony8980@163.com


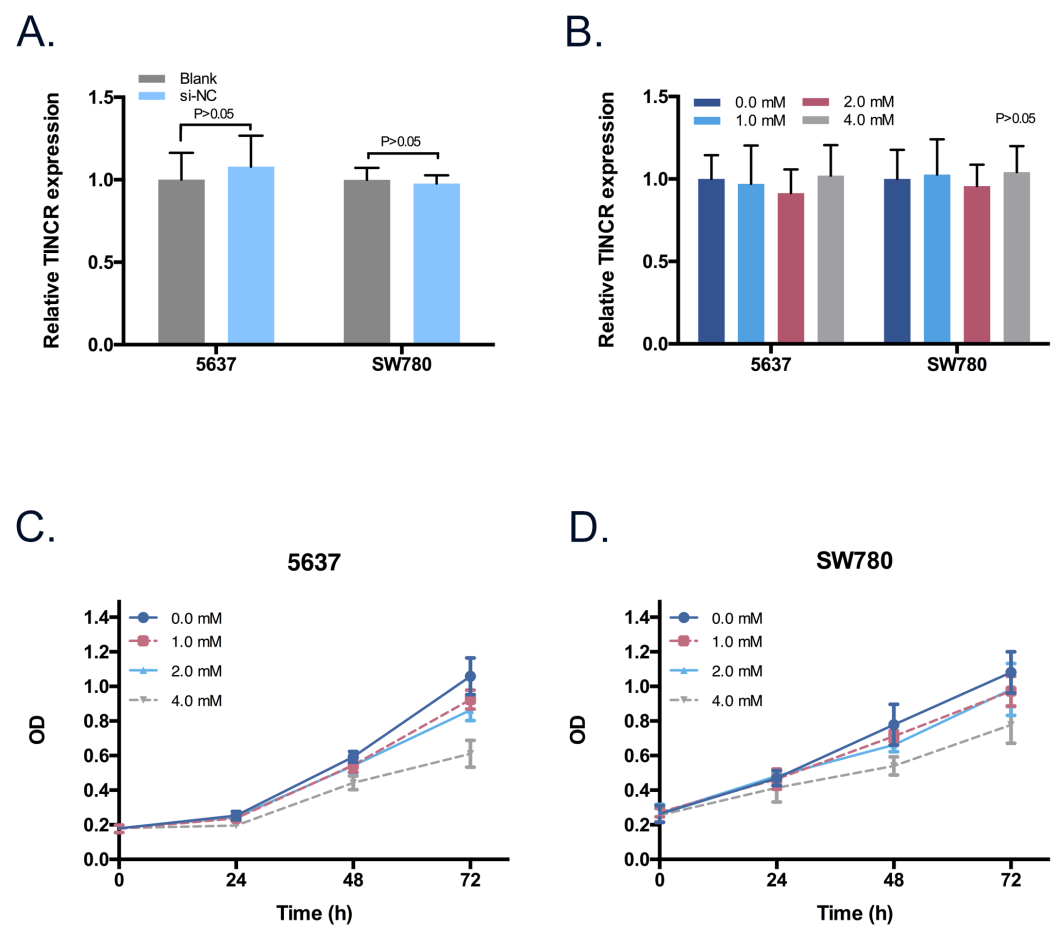


**Supplement 1**: (A) TINCR expression level following the treatment of 5637 and SW780 cells with si-NC and blank. (B) TINCR expression level of BCa cells at 0-4mM theophylline. P>0.05.(C, D) CCK8 assay demonstrated that 0-2mM theophylline have minimal effects on BCa cell viability. P>0.05.
